# Ascorbate-induced oxidative stress mediates TRP channel activation and cytotoxicity in human etoposide-sensitive and -resistant retinoblastoma cells

**DOI:** 10.1038/s41374-020-00485-2

**Published:** 2020-09-18

**Authors:** Jakub Oronowicz, Jacqueline Reinhard, Peter Sol Reinach, Szymon Ludwiczak, Huan Luo, Marah Hussain Omar Ba Salem, Miriam Monika Kraemer, Heike Biebermann, Vinodh Kakkassery, Stefan Mergler

**Affiliations:** 1 Klinik für Augenheilkunde, Charité – Universitätsmedizin Berlin, Corporate Member of Freie Universität Berlin, Humboldt-Universität zu Berlin and Berlin Institute of Health, Berlin, Germany; 2 Department of Cell Morphology and Molecular Neurobiology, Faculty of Biology and Biotechnology, Ruhr University Bochum, Bochum, Germany; 3 School of Ophthalmology and Optometry, Wenzhou Medical University, Wenzhou, PR China; 4 Institut für Experimentelle Pädiatrische Endokrinologie, Charité – Universitätsmedizin Berlin, Corporate Member of Freie Universität Berlin, Humboldt-Universität zu Berlin and Berlin Institute of Health, Berlin, Germany; 5 Universität zu Lübeck, Klinik für Augenheilkunde – Universitätsklinikum Schleswig-Holstein (Campus Lübeck), Lübeck, Germany

**Keywords:** Calcium signalling, Eye cancer, Patch clamp, Fluorescence imaging, Transient receptor potential channels

## Abstract

There are indications that pharmacological doses of ascorbate (Asc) used as an adjuvant improve the chemotherapeutic management of cancer. This favorable outcome stems from its cytotoxic effects due to prooxidative mechanisms. Since regulation of intracellular Ca^2+^ levels contributes to the maintenance of cell viability, we hypothesized that one of the effects of Asc includes disrupting regulation of intracellular Ca^2+^ homeostasis. Accordingly, we determined if Asc induced intracellular Ca^2+^ influx through activation of pertussis sensitive Gi/o-coupled GPCR which in turn activated transient receptor potential (TRP) channels in both etoposide-resistant and -sensitive retinoblastoma (WERI-Rb1) tumor cells. Ca^2+^ imaging, whole-cell patch-clamping, and quantitative real-time PCR (qRT-PCR) were performed in parallel with measurements of RB cell survival using Trypan Blue cell dye exclusion. *TRPM7* gene expression levels were similar in both cell lines whereas *TRPV1*, *TRPM2*, *TRPA1*, *TRPC5*, *TRPV4*, and *TRPM8* gene expression levels were downregulated in the etoposide-resistant WERI-Rb1 cells. In the presence of extracellular Ca^2+^, 1 mM Asc induced larger intracellular Ca^2+^ transients in the etoposide-resistant WERI-Rb1 than in their etoposide-sensitive counterpart. With either 100 µM CPZ, 500 µM La^3+^, 10 mM NAC, or 100 µM 2-APB, these Ca^2+^ transients were markedly diminished. These inhibitors also had corresponding inhibitory effects on Asc-induced rises in whole-cell currents. Pertussis toxin (PTX) preincubation blocked rises in Ca^2+^ influx. Microscopic analyses showed that after 4 days of exposure to 1 mM Asc cell viability fell by nearly 100% in both RB cell lines. Taken together, one of the effects underlying oxidative mediated Asc-induced WERI-Rb1 cytotoxicity stems from its promotion of Gi/o coupled GPCR mediated increases in intracellular Ca^2+^ influx through TRP channels. Therefore, designing drugs targeting TRP channel modulation may be a viable approach to increase the efficacy of chemotherapeutic treatment of RB. Furthermore, Asc may be indicated as a possible supportive agent in anti-cancer therapies.

## Abstract (web summary)

Ascorbate can act as an oxidant to induce tumor cell death at a pharmacological dose. Here the authors show that this response is associated with increases in GPCR Gi/o activity. This effect promotes rises in intracellular Ca^2+^ influx through transient receptor potential channel activity in retinoblastoma cells.

## Introduction

Retinoblastoma (RB) is the most common intraocular cancer solely expressed in children. It is the only central nervous system tumor that can be easily observed without dedicated medical equipment [1]. RB is due to a mutation of both RB1 alleles, which increases both formation of a phosphorylated protein product (pRB) and tumorous cell proliferation [2, 3]. In the absence of metastasis, the RB survival rate is variable in different countries. In developed countries, it has risen to over 90%, while it is <50% in lower-income countries having a higher incidence of RB patients [4, 5]. The first-line therapy in the worst cases is still enucleation despite numerous alternative options such as etoposide chemotherapy [3]. However, the efficacy of etoposide may be limited since some RB cells develop resistance [6]. Accordingly, there remains a need to develop novel approaches to treat this disease.

Dysfunctional regulation of intracellular calcium levels can disrupt control of responses that may underlie some types of RB neoplasms. In recent years, some progress was made in clarifying a relationship between alterations underlying tumorgenesis and dysfunctional transient receptor potential (TRP) channel expression [7–9]. One example includes an association between altered TRP channel expression and intracellular calcium regulation, which was recently described in healthy ocular cells and benign as well as malignant ocular tumor cells [10–12]. Sustained rises in intracellular calcium levels above 100 nM can lead to some damaging effects such as increases in apoptosis, autophagy, or even decreases in cell proliferation [13–16]. TRP channel involvement in regulating these responses suggests that they are potential drug targets to inhibit RB cell viability and survival.

The TRP channel superfamily is a heterogenous group of more than 28 channel genes that are divided into seven subfamilies based on protein and DNA sequence homology: TRPC (classical), TRPV (vanilloid), TRPM (melastatin), TRPA (ankyrin), TRPP (polycyclin), TRPML (mycolipin) and TRPN (NOMPC) [17–20]. RB cells express voltage-operated Ca^2+^ channels [21, 22] and TRPs [11, 23]. Through regulating Ca^2+^ influx, TRPs function as biosensors and transducers. They undergo polymodal activation in response to a wide variety of environmental stresses, including temperature fluctuation [24], tissue injury [25], anisoosmolarity [26], UV-light [27], pH reduction [28], certain ligands (e.g., capsaicin [29]), exocytosis, pathways coupled to phospholipase C stimulation and many others [30]. Changes in specific TRP expression levels and function are diagnostic of some malignant transformations [8, 31]. For example, either TRPV6 or TRPM8 upregulation is used as a marker for establishing a prostatic cancer prognosis [32, 33]. Therefore, identifying TRP channel expression patterns may broaden their use as prognostic tumor markers in various cancerous diseases [34].

Aside from acting as biosensors and transducers of a host of different environmental cues, TRP stimulation can also be triggered by the activation of G protein-coupled receptors (GPCRs), which are the largest family of signaling proteins in mammals [35, 36]. GPCRs and TRPs are both cell surface proteins of neuronal and non-neuronal cells. GPCRs and Ca^2+^ permeable TRPs are also a component of a GPCR-TRP signaling pathway axis and may function as a unit [37, 38].

Ascorbic acid (Asc) is being evaluated as a therapeutic option in treating oncologic diseases [39–41]. This treatment is becoming more widely used because of its beneficial effects in different groups of patients [42–46]. Pharmacological doses (i.e., 1–20 mM) of Asc administered through intravenous injection provide therapeutic benefit. In over 40 different tumor cell lines (including breast, lung, renal, ovarian cancer, and Y-79 RB cell line) Asc reduced cell survival [47, 48]. Even if a pharmacological dose of Asc administered as an adjuvant did not have a favorable outcome, millimolar levels of extracellular Vitamin C selectively killed cancer cells [49, 50] whereas these high dosages were well tolerated by healthy cells [41, 51].

Asc concentrations less than a millimolar are referred to as being physiological since they are similar to those reported in plasma (<0.2 mM). At these levels, Asc acts as an antioxidant. On the other hand, Asc at pharmacological doses is present in the plasma reaching the millimolar range in which case it acts instead as an oxidant generating hydrogen peroxide (H_2_O_2_), reactive oxygen species (ROS), and hydroxyl radicals [46, 47, 52]. In this study, 1 mM Asc was used, which is the same concentration used in a previous report [46].

Notably, Asc is present in human ocular tissues at millimolar levels. In this range, it is speculated that Asc protects these tissues from short-wavelength solar radiation damage [53]. Specifically, in ocular tissues the intracellular Asc concentration is reported as follows: lens: 2.5 to 3.4 mM; aqueous humour: 0.4–1.1 mM; corneal epithelium ~12,5 mM; retina > 1 mM [54, 55]. It is suggested that during intravenous ascorbate therapy Asc may reach comparable or even higher levels at the extracellular side of the retina through the ophthalmic artery. It is relevant because cell death depends on extracellular rather than the intracellular Asc concentration [49].

We show here that 1 mM Asc, presumably acting as an oxidant, had similar cytotoxic effects on etoposide-sensitive and etoposide-resistant WERI-Rb1 cell viability. This response included increases in Gi/o coupled GPCR activity resulting in increases in intracellular Ca^2+^ influx through TRP channels. The involvement of this channel family was confirmed based on (1) TRP gene expression patterns in the etoposide-sensitive and -resistant WERI-Rb1 cells; (2) Asc-induced increases in Ca^2+^ influx and underlying currents that are attributable to TRP channel activation; (3) association between the effects of TRP channel inhibitors and the two different aforementioned responses to Asc.

## Materials and methods

### Materials

Medium and supplements for cell culture were purchased from Biochrom AG (Berlin, Germany) or GIBCO Invitrogen (Karlsruhe, Germany). All reagents (e.g., Ascorbic acid A4403; Trypan Blue T8154), except those specified below, were purchased from Sigma-Aldrich (Deisenhofen, Germany). Capsazepine was purchased from Cayman Chemical Company (Ann Arbor, Mi, USA). The internal and external solutions for planar patch-clamping were provided by Nanion Company (Munich, Germany). Fura2/AM was purchased from PromoCell (Heidelberg, Germany).

### Cell culture and cultivation

**Fig. 1:**
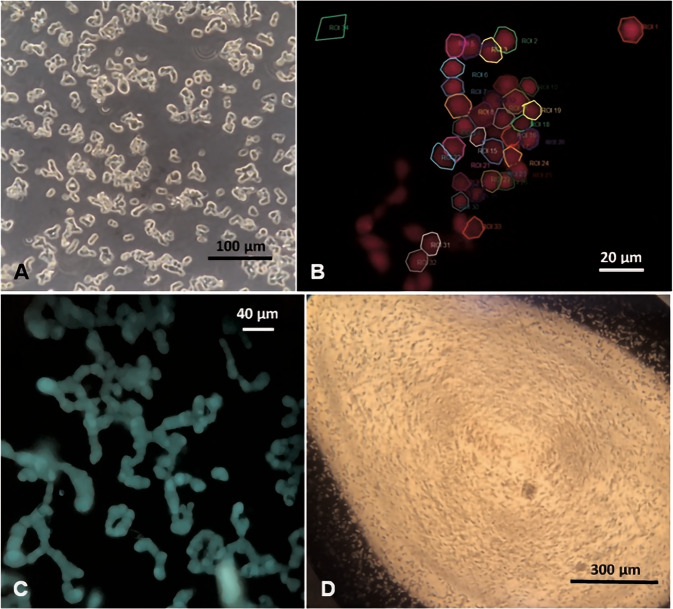
Microscopic images of RB cells. **a** Light microscopic image of etoposide-resistant WERI-Rb1 cells. **b** Fluorescence microscopic image (510 nm; red colored by imaging software) showing single etoposide-resistant WERI-Rb1 cells on a poly-L-lysine coated coverslip. The circumscribed zones point out some single cells that are regions of interest (ROIs) for fluorescence measurements. **c** Fluorescence microscopic image (510 nm) shows cells growing in chains. **d** Light microscopic view of a single-cell suspension prepared for patch-clamp recordings.

The etoposide-sensitive and -resistant WERI-Rb1 cell lines were established [6, 56, 57] and cultured as previously described [11, 58, 59]. In brief, both cell lines were cultivated in RPMI-1640 Medium [60], supplemented with 10% fetal bovine serum (FBS) and 100 IU/ml penicillin/streptomycin, in an incubator at 37 °C in 5% CO_2_ and 80% humidity. Cells were seeded on 12 well plastic culture plates (Fig. 1a) and medium was exchanged three times per week.

### Microscopic analyses of cell viability

The RB cells were prepared as described above. Subsequently, microscopic images were captured on the first, the fifth, and the seventh day. Two sets of experiments were performed in order to ascertain whether Asc altered viability of etoposide-resistant and -sensitive WERI-Rb1 cells. In the first set of experiments, freshly diluted cells were observed and 1 mM Asc was subsequently added to the medium. On the fifth day, cell density was again recorded and fresh RPMI-1640 medium was added to the cell culture to evaluate reversibility of perceived changes. On the seventh day, cell density images were captured and evaluated. In the second set of experiments, following culturing the cell suspensions for 4 days, 1 mM Asc was added to the medium. After another 4 days, the effect of this addition on cell density was reevaluated.

To ascertain if cell death accounted for the changes in their density, 0.4% Trypan Blue dye exclusion was used to evaluate cells viability. As only dead cells are stainable, on the fifth day 250 µl of the cell suspension were removed from each flask and diluted with an equal volume of Trypan Blue. With the Neubauer counting chamber (A. Hartenstein GmbH, Wuerzburg, Germany) stained cells were counted and expressed as a percent of the total cell population.

### RNA-isolation, cDNA synthesis, and qRT-PCR analysis

The Gene Elute Mammalian Total RNA Miniprep Kit from Sigma-Aldrich (St. Louis, USA) was used according to the manufacturer’s instructions to extract RNA from 5 × 10^6^ etoposide-sensitive and -resistant WERI-Rb1 cells. Concentration and purity of RNA were evaluated with a BioSpectrometer (Eppendorf, Hamburg, Germany). The First Strand cDNA Synthesis Kit was used for reverse-transcription of 1 µg RNA (Thermo Fisher Scientific, Waltham, MA, USA). qRT-PCR was performed using the FastStart essential DNA Green Master Mix. Reactions were run in a Light Cycler® 96 (Roche Applied Science, Mannheim, Germany). Conditions were as follows: 10 min at 95 °C; 10 s at 95 °C, 30 s at 60 °C; 10 s at 72 °C for 45 cycles; 10 s at 95 °C, 60 s at 65 °C, 1 s at 97 °C and 30 s at 37 °C. Oligonucleotides were designed using the ProbeFinder Assay Design Center (Roche Applied Science; Table 1). Efficiency of oligonucleotide pairs was calculated by a cDNA dilution series of 5 ng to 125 ng. For normalization and relative quantification, expression of the housekeeping gene *RPS18* was analyzed. Table 1 provides a list of oligonucleotides used for TRP-cation channel mRNA expression analyses of the aforementioned cell lines.

**Table 1:** List of oligonucleotides used for TRP-cation channel mRNA expression analyses in human etoposide-sensitive and -resistant WERI-Rb1 by qRT-PCR.

Gene	Oligonucleotide sequence	Product size (bp)	Oligonucleotide efficiency	GenBank accession number
*TRPA1*_for	TGGACACCTTCTTCTTGCATT	103	1.0	NM_007332
*TRPA1*_rev	TCATCCATTTCATGCAGCAC			
*TRPC5*_for	TGGTAACTGGTTCAACAACACC	99	1.0	NM_012471
*TRPC5*_rev	CTGTCAGCATTGCGTTCTG			
*RPS18*_for	CTTCCACAGGAGGCCTACAC	82	1.0	NM_022551
*RPS18*_rev	CGCAAAATATGCTGGAACTTT			
*TRPM2*_for	CGAGGACATCAGCAATAAGGT	75	0.836	NM_003307
*TRPM2*_rev	ATGGAGCCCGACCTCTTC			
*TRPM7*_for	TTGACATTGCCAAAAATCATGT	66	1.0	NM_017672
*TRPM7*_rev	CTTGTTCCAAGGATCCAACC			
*TRPM8*_for	GGTCCTGTACTCGCTGGTCT	67	1.0	NM_024080
*TRPM8*_rev	CACCCCATTTACGTACCACTG			
*TRPV1*_for	CTACAGCAGCAGCGAGACC	71	0.899	NM_080704
*TRPV1*_rev	CCTGCAGGAGTCGGTTCA			
*TRPV4*_for	CAACAACGACGGCCTCTC	74	1.0	NM_021625
*TRPV4_*rev	GGATGATGTGCTGAAAGATCC			

*bp* base pairs, *for* forward, *rev* reverse.

The oligonucleotide sequence, predicted product size, oligonucleotide efficiency, and GenBank accession number are indicated. For relative quantification of mRNA levels, *RPS18* served as reference gene.

### Fluorescence calcium imaging

Cells were preincubated with culture medium containing 1 µM Fura-2/AM and, if needed, with TRP-channel antagonists (for 20–30 min, except for N-acetylcysteine (NAC): 4–5 days) or Gi/o inhibitor—pertussis toxin (PTX) (for 18 h) at 37 °C and 5% CO_2_. Coverslips were additionally coated with poly-L-lysine to attach the RB cells. If dimethyl sulfoxide (DMSO) was used to dissolve a drug, its final concentration did not exceed 0.1%. At this concentration, it was not cytotoxic. Afterward, the cells were washed with a Ringer-like solution (containing in mM: 150 NaCl, 6 CsCl, 1 MgCl_2_, 10 HEPES acid, 10 glucose and 1.5 CaCl_2_ at pH ~7.35) in order to stop Fura-2/AM uptake and to remove any cell debris and dead cells [61]. Thereafter, fluorescence measurements were performed at room temperature ~20–23 °C using a digital imaging system (Olympus Europa Holding GmbH, Hamburg, Germany) in conjunction with an inverted microscope (Olympus BX50WJ), a LED light source (LED-Hub by Omikron, Rodgau–Dudenhoven, Germany) and a digital camera (Olympus XM-10). Alternate fluorescence wavelength excitation was isolated at 340 nm and 380 nm. Ratios (f_340_/f_380_) of emission at 510 nm were used to obtain relative values of intracellular Ca^2+^ levels with imaging software (cellSens, Olympus Europa Holding GmbH, Hamburg, Germany) (Fig. 1b, c) [62]. Drug application was done by pipetting it into the static measuring chamber. Results are shown as mean traces of the fluorescence ratio ± SEM (with bidirectional error bars). n*-*values indicate the number of measured cells. The fluorescence ratios f_340_/ f_380_ were normalized (control set to 0.1).

### Planar patch-clamp recordings

Following removal with phosphate buffer saline (PBS) of any cell debris to improve seal quality [63], a single-cell suspension was bathed in the external solution containing (in mM): 140 NaCl, 4 KCl, 1 MgCl_2_, 2 CaCl_2_, 5 D-glucose monohydrate and 10 HEPES (pH ≈ 7.4; osmolarity ≈ 298 mOsM) (Fig. 1d). Microchips (Nanion, Munich, Germany) with a mean resistance of 3–5 MOhm were used. First of all, the internal measuring solution was applied to the internal side of the chip (in mM): 50 CsCl, 10 NaCl, 60 CsF, 20 EGTA, and 10 HEPES (pH ≈ 7.2 and ≈ 288 mOsM). An external solution was then added to the external measuring side of the chip coupled to a planar patch-clamp system (Port-a-Patch®, Nanion, Munich, Germany), an EPC 10 amplifier (HEKA, Lamprecht, Germany), and PatchMaster version 2.6 for Windows (HEKA, Lamprecht, Germany) [64]. Furthermore, 5–10 µL of the single-cell suspension was added to the upper side of the microchip at ~20–23 °C. The software controlled the pump (Suction Control Pro, Nanion, Munich, Germany) in order to achieve and maintain the whole-cell configuration and also to read and evaluate the data. The recordings were started ~10 min after breaking into a whole-cell configuration and confirming the settings [65]. Currents were recorded every 5 s following a voltage ramp protocol of −60 to + 130 mV range without steps and 500 ms duration. Resulting currents were normalized to cell membrane capacitance to obtain current density (pA/pF). The software calculated mean access resistance (for etoposide-sensitive WERI-Rb1: 26 ± 3 MΩ; *n* = 19; for etoposide-resistant WERI-Rb1: 26 ± 3 MΩ; *n* = 16) and mean membrane capacitance (for etoposide-sensitive WERI-Rb1: 8 ± 2 pF; *n* = 19; for etoposide-resistant WERI-Rb1: 8 ± 2 pF; *n* = 16). The liquid junction potential was calculated (≈3.8 mV) and software corrected [66]. All current recordings were leak-subtracted. Only leak currents below 100 pA were used, while all other recordings were excluded from analyses. In order to avoid possible interference of voltage-dependent Ca^2+^ channels, the holding potential (HP) was set to 0 mV.

### Data analyses and statistics

Statistical significance was determined depending on whether the data passed the normality test. For data passing the normality test, paired or unpaired Student’s-t-test was used. Otherwise, the Wilcoxon test for matched pairs or Mann–Whitney *U* test for unpaired data were used. All other values are reported as means ± SEM. *P* values < 0.05 were considered as significant both for paired (*) and unpaired (#) tests. All analyses were performed using SigmaPlot version 12.5 for Windows (Systat Software, Inc., Point Richmond, California USA) and GraphPad Prism software version 5.00 for Windows (La Jolla, California, USA).

For statistical evaluation of qRT-PCR analysis, data were analyzed by REST 2009 (relative expression software tool; Qiagen GmbH, Hilden, Germany) using a pairwise fixed reallocation and randomization test. *P* values < 0.05 were considered statistically significant.

## Results

### *TRP* mRNA expression in etoposide-sensitive and -resistant WERI-Rb1 cells

**Fig. 2:**
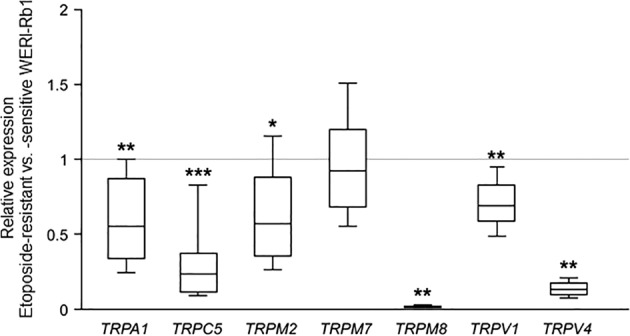
Analyses of relative TRP mRNA expression by qRT-PCR. Expression of TRPs was observed in etoposide-sensitive and -resistant WERI-Rb1 cells. Both cell lines show a comparable *TRPM7* mRNA expression. In contrast, *TRPA1*, *TRPC5*, *TRPM2*, *TRPM8*, *TRPV1,* and *TRPV4* mRNA levels were significantly downregulated in etoposide-resistant in comparison to the -sensitive WERI-Rb1 cells. Data are shown as median±quartile±minimum/maximum (*n* = 6).

Figure 2 provides a comparison of the gene expression levels of *TRPA1*, *TRPC5*, *TRPM2*, *TRPM7, TRPM8*, *TRPV1,* and *TRPV4* in etoposide-sensitive and -resistant WERI-Rb1 cells. Even though both cell lines had a comparable *TRPM7* mRNA expression level (0.920-fold; *p* = 0.556), *TRPC5* (0.239-fold; *p* < 0.001), *TRPM8* (0.015-fold; *p* = 0.001) as well as *TRPV4* (0.131-fold; *p* = 0.001) were markedly downregulated in the etoposide-resistant WERI-Rb1 cell line. In addition, *TRPA1* (0.556-fold; *p* = 0.002)*, TRPM2* (0.570-fold; *p* = 0.012) and *TRPV1* (0.687-fold; *p* = 0.001) expression levels were lower in the etoposide-resistant WERI-Rb1 cells. Overall, the gene expression levels of all of these TRPs were lower in etoposide-resistant WERI-Rb1 cell line, except for *TRPM7*.

### Asc induces larger Ca^2+^ increases in etoposide-resistant WERI-Rb1 cells

**Fig. 3:**
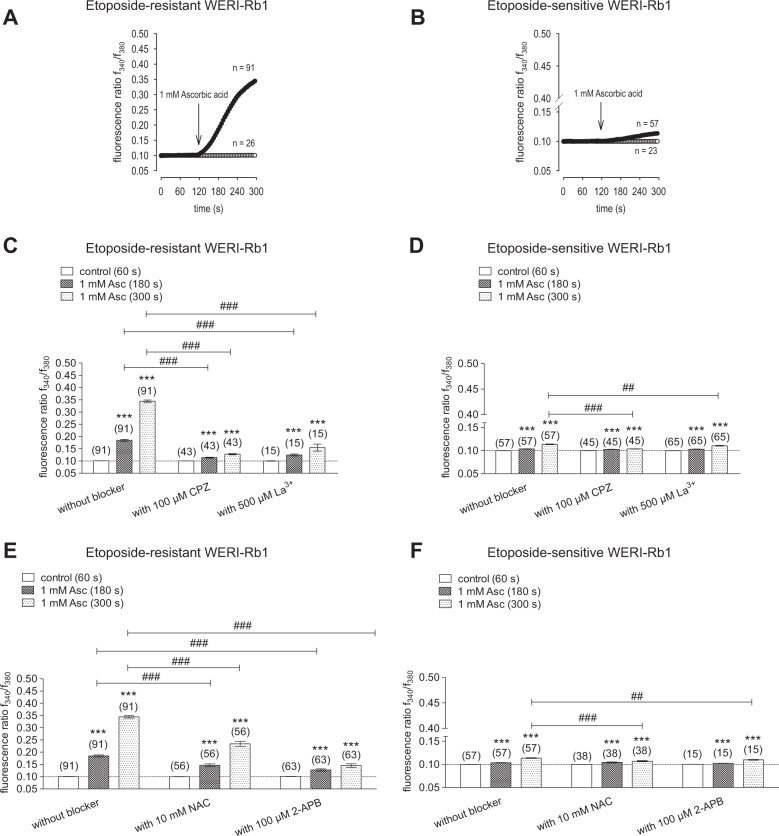
1mM Asc induces an increase in intracellular Ca^2+^ influx in both RB cell lines. TRP-channel antagonists (CPZ, La^3+^, NAC, and 2-APB) suppress Asc-induced Ca^2+^ influx. **a** 1 mM Asc led to an increase in intracellular Ca^2+^ influx (*n* = 91) in etoposide-resistant WERI-Rb1 cells, whereas non-treated control cells maintained a constant Ca^2+^ baseline (*n* = 26). **b** The same experiment as shown in (**a**) but carried out with etoposide-sensitive WERI-Rb1 cells. **c** Summary of the experiments with 1 mM Asc and TRP-channel antagonists (100 µM CPZ, 500 µM La^3+^) in etoposide-resistant WERI-Rb1 cells. The asterisks (*) designate a significant increase in fluorescence ratios (f_340/380_) in the groups of cells with and without TRP-channel antagonists after addition of Asc to the medium (paired tested). The hashtags (#) indicate significant differences in fluorescence ratios (f_340/380_) (unpaired tested). **d** Summary of the same experiments as in (**c**) but with etoposide-sensitive WERI-Rb1 cells. **e** Summary of the experiments with 1 mM Asc and TRP-channel antagonists (10 mM NAC and 100 µM 2-APB) in etoposide-resistant WERI-Rb1 cells. The asterisks (*) designate a significant increase in fluorescence ratios (f_340/380_) in the groups of cells with and without TRP-channel antagonists after addition of Asc to the medium (paired tested). The hashtags (#) indicate significant differences in fluorescence ratios (f_340/380_) (unpaired tested). **f** Summary of the same experiments as in (**e**) but with etoposide-sensitive WERI-Rb1 cells (unpaired tested only at *t* = 300 s). Asc Ascorbic acid, CPZ Capsazepine, La^3+^ Lanthanum-III-chloride, NAC N-acetylcysteine, 2-APB 2-aminoethyl diphenylborinate.

Asc (1 mM) induced larger increases in the f_340_/f_380_ fluorescence ratio in etoposide-resistant WERI-Rb1 cells than in their etoposide-sensitive counterpart. It increased the ratio in etoposide-resistant WERI-Rb1 cells from 0.1008 ± 0.0002 (*t* = 60 s; *n* = 91) to 0.3446 ± 0.0048 (*t* = 300 s; *n* = 91; *p* < 0.0001) (Fig. 3a, c), while in an etoposide-sensitive WERI-Rb1 group the fluorescence ratio increased only from 0.0998 ± 0.0001 (*t* = 60 s; *n* = 57) to 0.1135 ± 0.0008 (*t* = 300 s; *n* = 57; *p* < 0.0001) (Fig. 3b, d).

### TRP-channel antagonists suppress Asc-induced Ca^2+^ influx

The contribution by TRP channel activation to Asc-induced rises was evaluated based on comparing the inhibitory effects of various modulators of this response. Both RB cell lines were individually pretreated with either 100 µM capsazepine (CPZ), 500 µM Lanthanum-III-chloride (La^3+^), 10 mM n-acetyl cysteine (NAC), or 100 µM 2-aminoethyl diphenylborinate (2-APB). The results shown in Fig. 3c–f indicate that each of these inhibitors reduced Asc-induced increases in Ca^2+^ influx in both RB cell lines. Specifically, CPZ preincubation reduced the Asc-induced influx to 0.1278 ± 0.0026 (*t* = 300 s; *n* = 43; *p* < 0.0001) in etoposide-resistant WERI-Rb1 cells (Fig. 3c) and to 0.1036 ± 0.0004 (*t* = 300 s; *n* = 45; *p* < 0.0001) in etoposide-sensitive WERI-Rb1 cells (Fig. 3d). La^3+^ decreased the fluorescence ratios (f_340_/f_380_) in etoposide-resistant WERI-Rb1 cells to 0.1548 ± 0.0142 (*t* = 300 s; *n* = 15; *p* < 0.0001) and in etoposide-sensitive WERI-Rb1 cells to 0.1104 ± 0.0007 (*t* = 300 s; *n* = 65, *p* < 0.005), respectively (Fig. 3c, d). With NAC both RB cell lines were preincubated for 4–5 days, whereas preincubation time with the other TRP channel blockers was only 20–30 min [67]. This time for NAC is the longest possible because after a longer period cell viability declined, what affected measuring conditions. NAC also reduced the fluorescence ratios, in etoposide-resistant WERI-Rb1cells to 0.2344 ± 0.0103 (*t* = 300 s; *n* = 56; *p* < 0.0001) (Fig. 3e) and in etoposide-sensitive WERI-Rb1 cells to 0.1068 ± 0.0012 (*t* = 300 s; *n* = 38; *p* < 0.0001) (Fig. 3f). Preincubation with 2-APB reduced the fluorescence ratios to 0.1453 ± 0.0071 (*t* = 300 s; *n* = 63; *p* < 0.0001) in etoposide-resistant WERI-Rb1 cells (Fig. 3e) and to 0.1098 ± 0.0009 (*t* = 300 s; *n* = 15; *p* < 0.05) in etoposide-sensitive WERI-Rb1 cells (Fig. 3f). The inhibition of Asc-induced increases in intracellular Ca^2+^ levels shows that TRP channel activity contributes to these rises.

### PTX suppresses Asc-induced Ca^2+^ influx

**Fig. 4:**
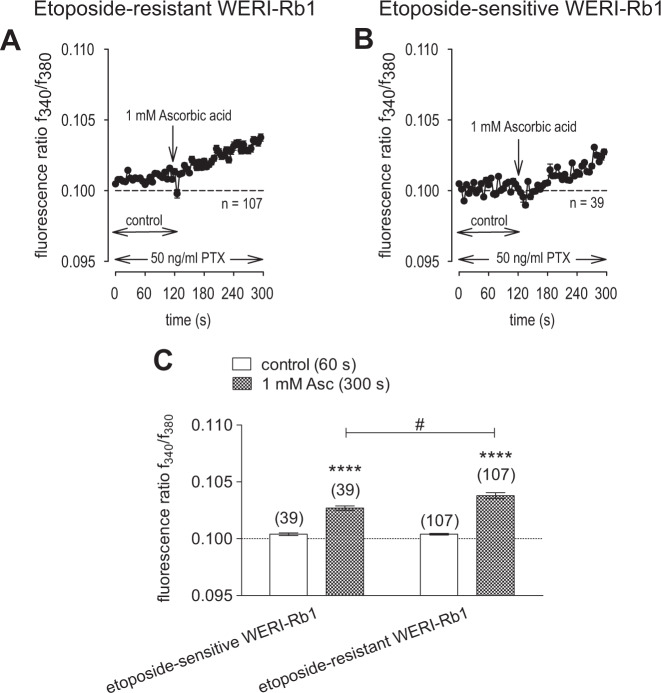
PTX suppresses Asc-induced Ca^2+^ influx in both RB cell lines. **a** Mean trace of etoposide-resistant WERI-Rb1 cells after addition of 1 mM Asc to the medium bathing cells pretreated with 50 ng/ml PTX (*n* = 107) for 18 h. **b** Mean trace of etoposide-sensitive WERI-Rb1 cells after addition of 1 mM Asc to the medium bathing cells pretreated with 50 ng/ml PTX (*n* = 39). **c** Summary of Asc experiments in the presence of PTX in etoposide-sensitive and -resistant WERI-Rb1 cells. The asterisks (*) designate a significant increase in fluorescence ratios (f_340/380_) after addition of Asc to the medium bathing each group of cells (paired tested) (*t* = 300 s). The hashtag (#) indicates a significant difference in fluorescence ratios (f_340/380_) between both RB cell lines (unpaired tested). PTX Pertussis toxin.

Since one of the effects of Asc is modulation of aminergic GPCRs in some cell types, both RB cell lines were preincubated with PTX (50 ng/ml) for 18 h to determine if Gi/o modulation by Asc contributes to controlling Ca^2+^ influx [68–71]. PTX preincubation blunted the Asc-induced rise in Ca^2+^ influx in both cell lines compared to untreated cells (Figs. 3a, b and 4a, b). Specifically, the fluorescence ratio in etoposide-resistant WERI-Rb1 only slightly increased from 0.1004 ± 0.0001 (*t* = 60 s) to 0.1038 ± 0.0003 (*t* = 300 s; *n* = 107; *p* < 0.0001) (Fig. 4a, c). Similarly, this ratio increased only to 0.1027 ± 0.0002 (*t* = 300 s; *n* = 39; *p* < 0.0001) in etoposide-sensitive WERI-Rb1 cells (Fig. 4b, c). Taken together, inactivation of Gi/o proteins nearly completely suppressed the Asc-induced activation of TRPs in both RB cell lines. This result suggests that Asc-induced stimulation of TRP activity is mediated through enhancement of a Gi/o coupled GPCR.

### Asc-induced increases in intracellular Ca^2+^ levels depend on extracellular Ca^2+^

**Fig. 5:**
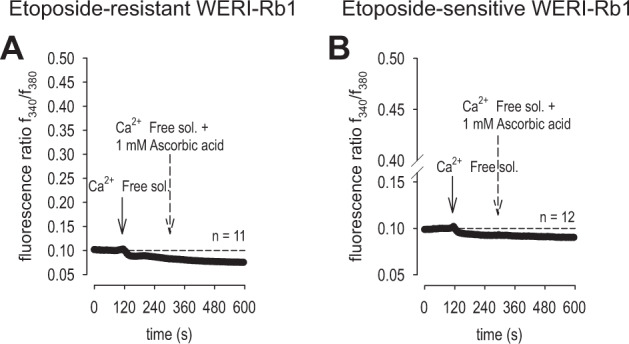
Asc-induced intracellular Ca^2+^ increase depends on extracellular Ca^2+^ in the bathing medium. **a** Substitution of Ca^2+^ containing medium with Ca^2+^-free solution led to a slight decrease of intracellular Ca^2+^ level in etoposide-resistant WERI-Rb1 cells (*n* = 11). Notable, addition of 1 mM Asc failed to increase the intracellular Ca^2+^ concentration as it took place in the presence of extracellular Ca^2+^. **b** The same experiment as shown in (**a**), but carried out with etoposide-sensitive WERI-Rb1 cells (*n* = 12).

To assess the source of Ca^2+^ in mediating the Asc-induced increases in intracellular Ca^2+^ levels, the effects of Asc on this response were determined in a Ca^2+^-free bathing solution containing 1 mM EGTA. This substitution decreased the fluorescence ratio to 0.0865 ± 0.0018 (*t* = 60 s before Asc addition; *n* = 11) in etoposide-resistant WERI-Rb1 cells. 1 mM Asc supplementation instead decreased it further to 0.0766 ± 0.0021 (*t* = 180 s after Asc addition; *n* = 11; *p* < 0.0001) (Fig. 5a). These declines were essentially the same in the etoposide-sensitive WERI-Rb1 cells. Namely, replacing the extracellular Ca^2+^ containing solution with the Ca^2+^-free bathing solution, the fluorescence ratio decreased to 0.0924 ± 0.0013 (*t* = 60 s before Asc addition; *n* = 12). Afterward, 1 mM Asc supplementation even further decreased this ratio to 0.0909 ± 0.0014 (*t* = 180 s after Asc addition; *n* = 12; *p* < 0.0001) (Fig. 5b). Taken together, these opposing effects of Asc in the absence of extracellular Ca^2+^ indicate that this oxidant induces rises in Ca^2+^ influx from the exterior through plasma membrane delimited pathways.

### Asc induces increases in whole-cell currents

**Fig. 6:**
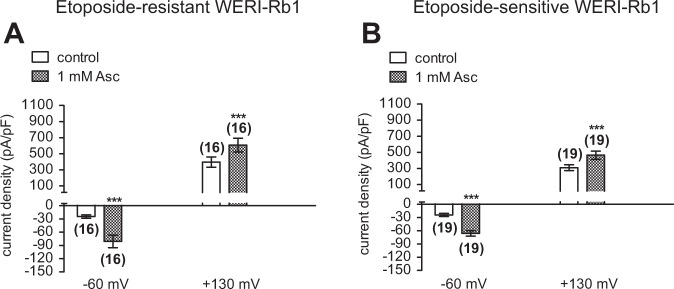
1mM Asc induces increases in whole-cell currents in both etoposide-resistant and -sensitive WERI-Rb1 cells. **a** Summary of patch-clamp experiments with Asc in etoposide-resistant WERI-Rb1 cells (*n* = 16). The asterisks (*) indicate statistically significant increase in whole-cell currents after application of 1 mM Asc (paired tested). **b** Same summary as in (**a**) except etoposide-sensitive WERI-Rb1 cells are instead analyzed (*n* = 19). Asc Ascorbic acid.

To confirm that 1 mM Asc-induced increases in Ca^2+^ influx are mainly reflective of the rises in ion channel currents, its effects were determined on whole-cell currents induced by applying a voltage ramp from −60 to + 130 mV, in both etoposide-resistant and -sensitive WERI-Rb1 cells. Specifically, in the etoposide-resistant WERI-Rb1 cells, the inward current density increased from −25 ± 2 pA/pF to −81 ± 8 pA/pF (*n* = 16; *p* < 0.0005). Similarly, the outward currents increased from 397 ± 38 pA/pF to 529 ± 51 pA/pF (*n* = 16; *p* < 0.0001) (Fig. 6a). Comparable results were observed in a group of etoposide-sensitive WERI-Rb1 cells. The inward current density increased from −24 ± 2 pA/pF to −66 ± 4 pA/pF (*n* = 19; *p* < 0.0001), while the outward currents increased markedly from 309 ± 23 pA/pF to 464 ± 34 pA/pF (*n* = 19; *p* < 0.0005) (Fig. 6b). Interestingly, there were no significant differences in the whole-cell current densities between both cell lines. These results confirm that Asc-induced increases in whole-cell currents underlie rises in Ca^2+^ influx.

### TRP-channel antagonists suppress Asc-induced increases in whole-cell currents

**Fig. 7:**
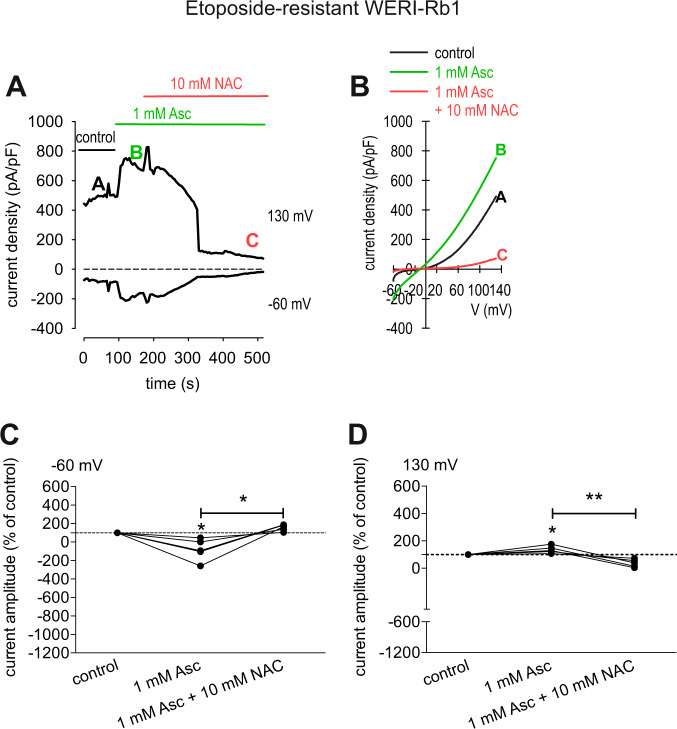
NAC blocks the Asc-induced increase in whole-cell currents in etoposide-resistant WERI-Rb1 cells. **a** Time course recording of the currents increase induced by 1 mM Asc and currents decrease after application of 10 mM NAC. **b** Original traces of Asc-induced current responses to voltage ramps [current/voltage plot (I-V plot)]. Current densities are shown as a control without drugs (black trace labeled as A), during application of 1 mM Asc (green trace labeled as B) and after addition of 10 mM NAC (red trace labeled as C). Calculated current densities obtained by normalizing currents to membrane capacitance as function of imposed voltage were derived from the traces shown in (**a**). **c** Maximal negative inward current amplitudes induced by a voltage step from 0 mV to −60 mV, shown in percent of control values before application of drugs (control set to 100%). The asterisks (*) designate a significant increase in inward currents after application of 1 mM Asc and a significant suppression of inward currents after addition of 10 mM NAC (paired tested). **d** Same diagram but related to maximal outward current amplitudes induced by a voltage step from 0 mV to +130 mV. Asc Ascorbic acid, NAC N-acetylcysteine.

**Fig. 8:**
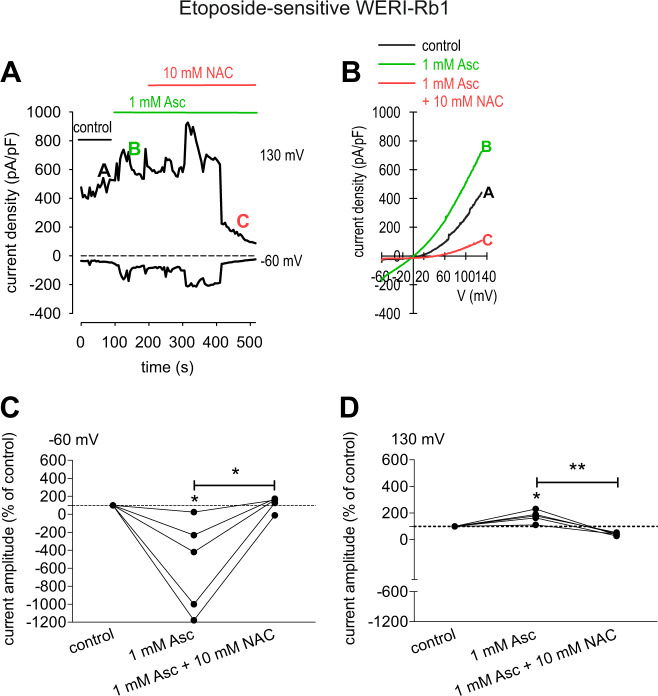
NAC blocks the Asc-induced increase in whole-cell currents in etoposide-sensitive WERI-Rb1 cells. **a** Time course recording of the currents increase induced by 1 mM Asc and currents decrease after application of 10 mM NAC. **b** Original traces of Asc-induced current responses to voltage ramps [current/voltage plot (I-V plot)]. Current densities are shown as a control without drugs (black trace labeled as A), during application of 1 mM Asc (green trace labeled as B) and after addition of 10 mM NAC (red trace labeled as C). Calculated current densities obtained by normalizing currents to membrane capacitance as function of imposed voltage were derived from the traces shown in (**a**). **c** Maximal negative inward current amplitudes induced by a voltage step from 0 mV to -60 mV, shown in percent of control values before application of drugs (control set to 100%). The asterisks (*) designate a significant increase in inward currents after application of 1 mM Asc and a significant suppression of inward currents after addition of 10 mM NAC (paired tested). **d** Same diagram but related to maximal outward current amplitudes induced by a voltage step from 0 mV to +130 mV. Asc Ascorbic acid, NAC N-acetylcysteine.

**Fig. 9:**
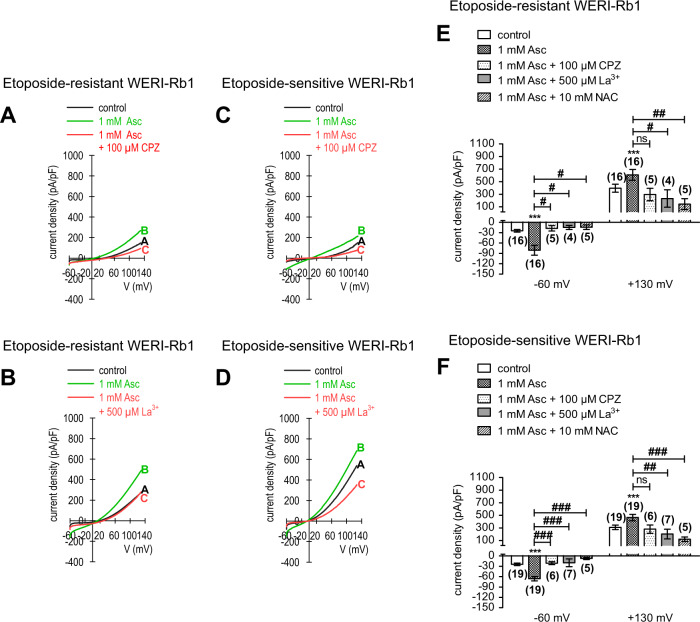
TRP antagonists (CPZ, LA^3+^, NAC) can block Asc-induced increase in whole-cell currents in both etoposide-resistant and -sensitive WERI-Rb1 cells. **a** Original traces of Asc-induced current responses to voltage ramps [current/voltage plot (I-V plot)] in etoposide-resistant WERI-Rb1 cells. Current densities are shown as a control without drugs (labeled as A), during application of 1 mM Asc (labeled as B) and after addition of 100 µM CPZ (labeled as C). **b** The same experiment as in (**a**), but with 500 µM La^3+^ in etoposide-resistant WERI-Rb1 cells. **c** Same experiment as in (**a**) but with etoposide-sensitive WERI-Rb1 cells. **d** Same experiment as in (**b**) but with etoposide-sensitive WERI-Rb1 cells. **e** Summary of patch-clamp experiments with Asc and TRP-channel antagonists (CPZ, La^3+^, NAC) in etoposide-resistant WERI-Rb1. The asterisks (*) indicate a significant increase in whole-cell currents after application of 1 mM Asc (paired tested). The hashtags (#) designate significant decreases in whole-cell currents after adding TRP-channel antagonists (100 µM CPZ, 500 µM La^3+^, 10 mM NAC) (unpaired tested). ns not significant (unpaired tested). **f** Same summary as in (**e**) but concerning etoposide-sensitive WERI-Rb1 cells. Asc Ascorbic acid, CPZ Capsazepine, La^3+^ Lanthanum-III-chloride, NAC N-acetylcysteine.

TRP channel inhibitors were used at the same concentration as those in the Ca^2+^ imaging experiments, except for 2-APB that was not used. Each of them suppressed whole-cell currents in both etoposide-resistant and -sensitive WERI-Rb1 cells. NAC suppressed the Asc-induced rises in inward currents in the etoposide-resistant WERI-Rb1 cells from −125 ± 11 pA/pF to −14 ± 1 (*n* = 5; *p* < 0.05), while the outward currents markedly decreased from 810 ± 70 pA/pF to 144 ± 13 pA/pF (*n* = 5; *p* < 0.05) (Figs. 7 and9e). Similar results were obtained in etoposide-sensitive WERI-Rb1 cells. In this cell line, the inward current density decreased from −72 ± 3 pA/pF to −7 ± 0 pA/pF (*n* = 5; *p* < 0.0005), while the outward currents fell from 537 ± 34 pA/pF to 121 ± 5 pA/pF (*n* = 5; *p* < 0.005) (Figs. 8 and 9f). CPZ had similar effects since the inward current density in etoposide-resistant WERI-Rb1 cells declined from −69 ± 7 pA/pF to −18 ± 3 pA/pF (*n* = 5; *p* < 0.05) along with the outward currents, which decreased from 652 ± 42 pA/pF to 296 ± 33 pA/pF (*n* = 5; *p* > 0.05) (Fig. 9a, e). Similarly, in etoposide-sensitive WERI-Rb1 the inward current density decreased from −57 ± 5 pA/pF to −21 ± 2 pA/pF (*n* = 6; *p* < 0.005), which was accompanied by declines in the outward currents from 394 ± 30 pA/pF to 281 ± 24 pA/pF (*n* = 6; *p* > 0.05) (Fig. 9c, f). With La^3+^, the inward currents in etoposide-resistant WERI-Rb1 cells decreased from −61 ± 5 pA/pF to −15 ± 1 pA/pF (*n* = 4; *p* < 0.05) along with the outward currents, which fell from 451 ± 25 pA/pF to 233 ± 21 pA/pF (*n* = 4; *p* < 0.05) (Fig. 9b, e). Also, in etoposide-sensitive WERI-Rb1 cells, the inward current density decreased from −63 ± 3 pA/pF to −20 ± 2 pA/pF (*n* = 7; *p* < 0.0005). The outward currents were suppressed from 414 ± 32 pA/pF to 203 ± 12 (*n* = 7; *p* < 0.005) in the presence of La^3+^ (Fig. 9d, f). These inhibitory effects confirm that Asc-induced TRP channel activation contributes to increases in Ca^2+^ influx.

### Medium acidification has less influence on Ca^2+^ regulation

**Fig. 10:**
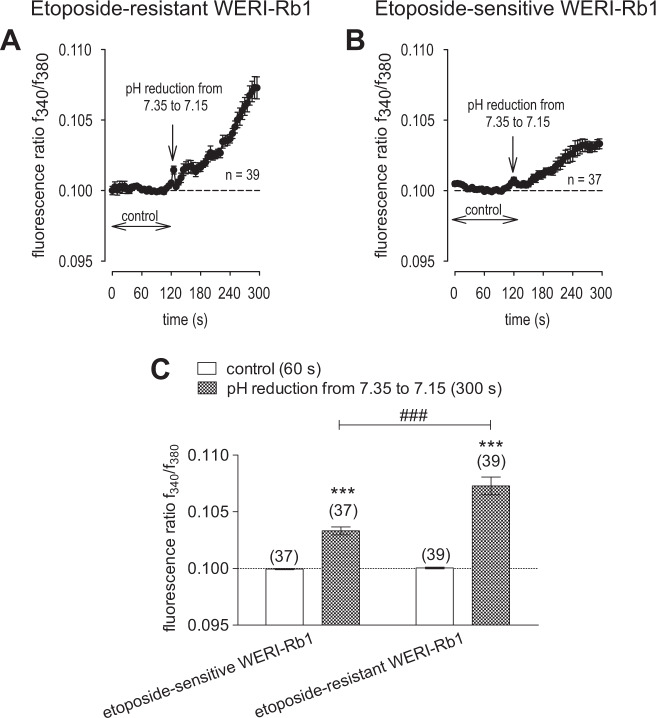
Medium acidification has less influence on Ca^2+^ regulation. **a** Mean trace of etoposide-resistant WERI-Rb1 cells under pH reduction (i.e., from 7.35 to 7.15) (*n* = 39). **b** Mean trace of etoposide-sensitive WERI-Rb1 cells under pH reduction (i.e., from 7.35 to 7.15) (*n* = 37). **c** Summary of pH reduction experiments compared to control values of etoposide-sensitive and -resistant WERI-Rb1 cells. pH reduction induced larger Ca^2+^ influx in etoposide-resistant WERI-Rb1 cells. The asterisks (*) designate a significant increase in fluorescence ratios (f_340/380_) after pH reduction in each group of cells (paired tested) (*t* = 300 s). The hashtags (#) indicate significant differences in fluorescence ratios (f_340/380_) between both RB cell lines (unpaired tested).

As medium acidification is one of the factors that can induce TRP channel activation, we determined if such a change contributes to how Asc stimulates TRPs. This possibility warranted consideration since 1 mM Asc decreased the bathing solution pH from ~7.35 to ~7.15, at room temperature ~20–23 °C. To replicate such a decline, we determined if merely acidifying the bathing solution with HCl also increased the fluorescence ratio f_340_/f_380_ in RB cells. Indeed, the fluorescence level slightly rose from 0.1004 ± 0.0001 to 0.1073 ± 0.0008 (*t* = 300 s; *n* = 39; *p* < 0.0001), in etoposide-resistant WERI-Rb1 cells. Similarly, this ratio increased from 0.0999 ± 0.0001 to 0.1033 ± 0.0004 (*t* = 300 s; *n* = 37; *p* < 0.0001) in etoposide-sensitive WERI-Rb1 cells (Fig. 10). Taken together, minor medium acidification slightly increased intracellular Ca^2+^, but the effect is too small to fully account for how Asc stimulates TRPs.

### Asc suppresses RB cell viability

**Fig. 11:**
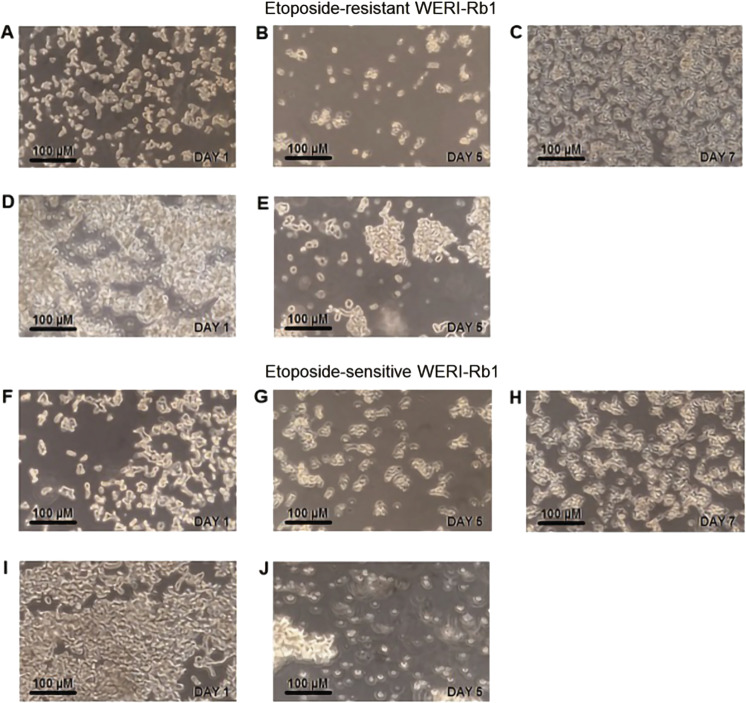
Effects of exposure to Asc on RB cell viability (a–e—etoposide-resistant WERI-Rb1 cells; f–j—etoposide-sensitive WERI-Rb1 cells). **a** Microscopic image of freshly diluted cells on the first day. Subsequently, 1 mM Asc was added. **b** The same cells as shown in (**a**), but on the fifth day after Asc treatment. A clear reduction of cell density is visible. Subsequently, medium dilution with fresh RPMI-1640 medium. **c** The same cells as in (**a**) and (**b**), but 2 days after adding fresh RPMI-1640 medium. The cell density again increased. **d** Microscopic image showing etoposide-resistant WERI-Rb1 cells which were maintained for 4 days in culture. At this time, 1 mM Asc was added. **e** The same cells as in (**d**), but on the fifth day after Asc treatment. The cell density declined. **f–j** The same experimental design as that shown in (**a**–**e**), but etoposide-sensitive WERI-Rb1 cells were used. Similar to the etoposide-resistant WERI-Rb1 cells, cell density declined after Asc treatment and recovered after diluting the medium.

**Fig. 12:**
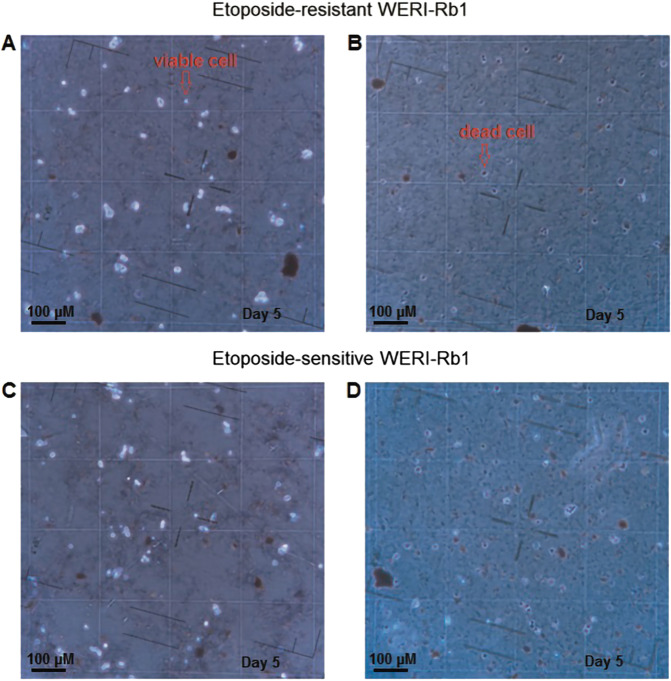
1mM Asc suppresses RB cell viability. Trypan Blue dye exclusion capability is compared between control and Asc-exposed WERI-Rb1 cells, on the fifth day after Asc treatment. In the background, is shown the cell counting chamber divided into square fields. **a** Light microscopic image of etoposide-resistant WERI-Rb1 cells. Most cells are viable (e.g., marked with a red arrow)—excluded the Trypan Blue dye. **b** The same group of cells as in (**a)**, but exposed to 1 mM Asc. Most cells are dead (e.g., marked with a red arrow)—Trypan Blue dye stains cell interior. **c**, **d** These panels show the same conditions as in (**a**, **b**) except that a group of etoposide-sensitive WERI-Rb1 cells are shown instead.

The cell culture images shown in Figs. 11 and 12 are indicative of the cytotoxic effects of 1 mM Asc in both the etoposide-sensitive and -resistant WERI-Rb1 cell groups. After 4 days of incubating freshly diluted cells with Asc, the cell densities declined in both types of RB cells (Fig. 11a, b, f, g). Comparing Fig. 11b vs. d and Fig. 11g vs. i shows that the cell densities fell compared to those in untreated cells. Diluting the medium with fresh RPMI-1640 medium led to a recovery of the cell density. Interestingly, these rescued cells failed to cluster together to form any compact groups (Fig. 11b, c, g, h). In the second set of experiments, 1 mM Asc was added to the medium 4 days after the cells were passaged. Subsequently, these clumped groups separated during the time when the cell density progressively declined (Fig. 11d, e, i, j).

To determine if declines in cell density are reflective of losses in cell viability, Trypan Blue dye exclusion was used to evaluate the viability of RB cells after 4 days in culture (in the first set of experiments) (Fig. 12). In etoposide-resistant WERI-Rb1, the percentage of dead cells increased from 6.1 ± 0.6% (*n* = 8) in the untreated group shown in Fig. 12a (analogical to Fig. 11d) to 93.7 ± 1.5% (*n* = 16) (*p* = 0.0001) in the Asc-treated counterpart shown in Fig. 12b (analogical to Fig. 11b). Similarly, this percentage increased from 5.8 ± 1.0% (*n* = 8) to 93.9 ± 1.3% (*n* = 16) (*p* = 0.0001) in the etoposide-sensitive WERI-Rb1 cells. This difference is evident by comparing the results shown in Fig. 12c, d (analogical to Fig. 11i, g).

In summary, 1 mM Asc led to the cell death as indicated by the declines in the density of etoposide-resistant and etoposide-sensitive WERI-Rb1 cells, which was partially reversible upon dilution with fresh medium.

## Discussion

In agreement with numerous studies in other cell lines, we show here that a 1 mM pharmacological dose of Asc had similar cytotoxic effects on both etoposide-resistant and -sensitive WERI-Rb1 cells through acting allegedly as an oxidant [44, 47, 50]. The results indicate that these declines in cell viability are mediated through increases in Gi/o activity which in turn stimulate TRP channels resulting in increases in intracellular Ca^2+^ influx. At this concentration, it is presumed that Asc acts as prooxidant and generates H_2_O_2_ and ROS as well as other oxidative species, which reduce cell viability [39, 54]. Therefore, our results provide for the first time some insight into an interaction between Gi/o coupled GPCR and TRP activity in mediating Asc-induced cytotoxicity in both etoposide-sensitive and -resistant WERI-Rb1 cells.

A pattern of TRP channel gene expression was identified that agrees with a previously identified grouping in both WERI-Rb1 cell lines (i.e., Fig. 2) [11, 23]. Since both a battery of a relatively selective TRP channel antagonists combined with broad spectrum Ca^2+^ channel blockers inhibited Asc-induced rises in Ca^2+^ influx, activation of different TRP channel subtypes contributes to this response in RB cells (Fig. 3). Asc failed to increase such influx during exposure to PTX, suggesting that TRP channel activation is dependent on Asc interaction with the Gi/o coupled GPCR proteins (Fig. 4).

It is already known that excessive uncompromised rises in intracellular Ca^2+^ levels can lead to cell death or deregulate cancerogenic pathways [72, 73]. However, the identity of the mechanisms mediating such control still requires clarification. This study revealed for the first time that TRP channel activation on the cell membrane contributes to the Asc-induced Ca^2+^ influx in RB cells (Figs. 5–9). Mimicking with HCl addition the slight acidifying effect of Asc, it had a minor impact on intracellular Ca^2+^ levels. This suggests that a small decline in pH is not the sole mechanism accounting for how Asc induces TRP activation (Fig. 10). In the presence of Asc, RB cell density markedly declined due to inhibition of cell viability (Figs. 11 and 12). This cytotoxic effect of Asc agrees with similar studies reporting reduced viability of cells treated with ascorbate [47]. Interestingly, the Asc-induced Ca^2+^ increases in etoposide-resistant WERI-Rb1 cells were greater than those in etoposide-sensitive WERI-Rb1 cells. This difference suggests that Asc could be used as an anti-cancer adjuvant to overcome etoposide resistance that may be present in some types of cancerous growth.

### Role of Asc

Asc has numerous possible modes of action including mediating cellular redox reactions [50, 54]. In addition, Asc can directly modulate different receptors such as GABA [74]. Furthermore, Asc at concentrations of 1 mM or higher is able to inhibit tumor growth [75]. By acting as an oxidant, Asc generates H_2_O_2_ through reactions such as autooxidation [54]. Notably, H_2_O_2_ is the central redox molecule in oxidative stress [50, 76, 77] and was suggested to be the main factor that limited cell viability [39, 54]. Furthermore, cell death is dependent on extracellular but not intracellular Asc since this cytotoxic effect is mediated by extracellular H_2_O_2_ [49]. Interestingly, Chen et al. suggested that ascorbate radicals and H_2_O_2_ were present only in the extracellular fluid but were not detectable in whole blood [78]. The cell viability was also affected by intracellular GSH (glutathione) depletion, which increased oxidative stress [54] and by autophagy-associated caspase-independent cell death [79]. Moreover, Asc-induced oxidative stress can disrupt mitochondrial function [80]. Noteworthy is the finding that millimolar levels of extracellular Asc selectively induce tumor cell death [49, 50].

### Mechanisms of Asc-induced TRP-channel activation

We hypothesized that TRP channel subtypes are involved in mediating the cytotoxic effects of oxidant promoting Asc since some of them are sensitive to oxidative stress. They include: TRPA1 [81, 82], TRPV4 [83], TRPM7 [84], TRPM2, and TRPC5 [80, 85, 86]. Besides, TRPM7 channel modulation also affects RB cell growth [23]. Furthermore, changes in TRPV1 and TRPM8 expression levels were reported to play a significant role in tumorigenesis [87]. We investigated both functional and genetic expression of these channels in etoposide-resistant and -sensitive WERI-Rb1 cells. Accordingly, the effects were determined of the TRP channel antagonists with different specificity and selectivity, i.e., 100 µM CPZ that blocks TRPV1 and TRPM8 and 500 µM La^3+^ which is a broad spectrum Ca^2+^ channel blocker [88]. Moreover, CPZ can also potentiate the apoptotic effects of TRAIL (TNF-related apoptosis-inducing ligand) via ROS-JNK-CHOP pathway or downregulate the nuclear transcription factor-kappa B (NF-κB) [89, 90]. The non-selective inhibitors included also 2-APB (100 µM) which blocks TRPC5 and presumably TRPM2 as well as store operated calcium channel entry [67, 91–93]. Another broad-spectrum antagonist was 10 mM NAC, which blocks TRPA1, TRPV1, and TRPM2 [67, 94–96]. Besides, NAC has this classification, because it can also quench free radicals and impact GSH regeneration [96]. As several reports suggested using a longer pretreatment period [67, 95], we incubated the RB cells for up to 5 days with NAC rather than ~20–30 min, which was the incubation time for all the other antagonists. This was the longest period that did not reduce cell viability and did not impact measuring conditions. As the inhibitory effects of NAC were at some times larger than those of other inhibitors, it is conceivable that Asc induces rises in Ca^2+^ influx through additional pathways.

**Fig. 13:**
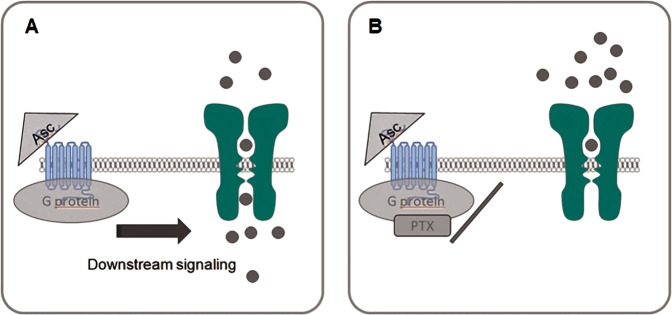
Schematic illustration of PTX effect on TRP channel. GPCRs (blue) and TRP channels (green) are often co-expressed in cells. It is accepted that activation of GPCRs modify the function of TRPs [37]. **a** Asc was reported as a modulator of aminergic GPCRs [68] and thereby might enhance the constitutive activity of these receptors. Therefore, downstream signaling effects might stimulate the activity of TRPs. **b** PTX is known to ADP-ribosylation of Gi/o proteins [71] that result in their inaction, thus reduce the activity of TRPs. Asc Ascorbic acid, PTX Pertussis toxin.

The effects of Asc were dependent on Gi/o interaction with TRP channels since preincubation with PTX inhibited Asc-induced increases in Ca^2+^ influx through these channels (Fig. 4). This dependence is in accord with other studies showing that GPCR activation triggers TRPs and vice versa (reviewed by Veldhuis et al. [37]). Furthermore, Asc is a well-characterized aminergic GPCR enhancer and many aminergic GPCRs are coupled to Gi/o [68] (Fig. 13).

The effects of Asc are dependent on its interaction with plasma membrane delimited channels since Asc had no effects on intracellular Ca^2+^ levels in an external Ca^2+^-free medium (Fig. 5). A similar dependence was reported for some other drugs [97].

Extracellular Asc application increased the inward and outward currents in both RB cell types. The correspondence between the Asc-induced increases in currents, Ca^2+^ transients, and the effects of the different blockers indicates that most of the current increases are attributable to a rise in Ca^2+^ influx (Figs. 6–9). Even though it is uncertain if 1 mM Asc induces these changes through acting as an oxidant, some studies show that increases in H_2_O_2_ generation induce TRP channel activation [98–102].

Since some TRPs are pH-sensitive and 1 mM Asc decreased the medium pH by 0.2 pH units (i.e., from ~7.35 to ~7.15), we determined if such an effect on pH by Asc accounts for TRP channel activation. Significant increases in Ca^2+^ influx were detected, but they were much smaller than those induced by 1 mM Asc (Fig. 10). Therefore, it is unlikely that these Ca^2+^ increases were induced by a decline in pH since a potentiating effect on TRP-induced mean current amplitude was reported to occur only if the medium pH was lowered below 6.0 [103–105]. In this case, these cell lines possibly possess other pH sensitive pathways since we still observed significant increases in Ca^2+^ influx. Interestingly, Garrity et al. also reported that TRP channel responses increased with decreasing pH, but these effects were probably caused by intracellular rather than by extracellular pH changes [106]. Therefore, pH acidification is unlikely to account for TRP channel activation since in this study the bathing solution pH was always >7.0.

It is noteworthy that there is a lack of correlation between the levels of functional TRP expression and corresponding gene expression levels in the two different cell types. In some other studies such a disconnect has been attributed to the possibility that mRNA turnover is variable due to modulation by other factors. Perhaps there is a closer correspondence instead between protein expression level changes and functional activity since protein expression turnover frequently occurs less rapidly than mRNA expression levels.

### RB cells viability under Asc incubation

Chen et al. suggested that Asc can affect the viability of breast, lung, renal, ovarian, and other cancer cells. They calculated the Asc concentration that was able to reduce cell survival by 50% [47]. Asc was also reported to affect the viability of Y-79 RB cell line [48]. In the current study, Trypan Blue dye exclusion was used to determine the effects of 1 mM Asc on cell viability. Such treatment decreased cell density in both etoposide-resistant and -sensitive WERI-Rb1 cells (Fig. 11a, b, f, g), compared to the untreated control groups (Fig. 11b vs. d; Fig. 11g vs i). Furthermore, Asc also dispersed preformed cell clusters (Fig. 11d, e, i, j). As shown in Fig. 11b, c, g, h, the reversal of declines in cell density may be attributable to a decrease in Asc concentration due to dilution of the medium and to the fact that after 5 days of Asc incubation about 6% of the cells retained viability in the cell culture (Fig. 12). The cytotoxic effects of a pharmacological dose of Asc could stem from increases in H_2_O_2_ generation, which was reported to promote cell death [49]. Such effects in another study induced apoptosis in podocytes [107]. Similarly, Asc-induced ROS-dependent apoptosis in thyroid cancer cells [108]. Comparable effects were obtained in breast cancer cells or retinal ganglion cells, independent from increases in H_2_O_2_ generation [109, 110]. Future studies are warranted to determine if Asc decreases RB cell viability through the same mechanisms described in a number of other different cancer cell types.

### Clinical relevance

Many studies described the possible therapeutic effects of Asc in different types of cancers [44, 46, 47, 49, 50, 54, 79, 111]. Nowadays, in an era of increasing numbers of cancer patients, more options are needed to improve therapeutic management of this disease.

In some clinical studies similar to those performed by Riordan et al., Asc is administered in a trial called “Riordan therapy” [39]. This group developed a protocol for administering a high intravenous Asc dose. It is likely that the Asc concentration is high enough for it to act as an oxidant rather than as a reductant. By acting as an oxidant, it promotes H_2_O_2_ generation and cytotoxicity, which is responsible for reducing cancer cell expansion [39, 54]. The current study is unique because it shows that Asc induces increase in cytotoxicity in RB cells through interacting with the Gi/o signaling pathway axis which in turn elicits increases in TRP channel activity followed by rises in intracellular Ca^2+^ influx. These results are relevant since TRPs were reported as an important factor in tumorigenesis [34, 112]. Furthermore, the current findings have an impact on gaining insight into how Ca^2+^ homeostasis is maintained and such control plays an important role in apoptosis and other cellular processes [13, 16, 113].

As already reported, targeting certain TRPs with either Asc or other drugs could serve as a main or supportive kind of cure in tumor diseases [114–118].

Since Asc-induced Ca^2+^ influx was larger in etoposide-resistant WERI-Rb1 cells than in the etoposide-sensitive WERI-Rb1 cells, Asc has the potential to serve as an adjuvant to improve the therapy of not only etoposide-sensitive but also cytostatic-resistant tumor cells.
