# Correction: Ascorbate-induced oxidative stress mediates TRP channel activation and cytotoxicity in human etoposide-sensitive and -resistant retinoblastoma cells

**DOI:** 10.1038/s41374-020-00499-w

**Published:** 2021-06-18

**Authors:** Jakub Oronowicz, Jacqueline Reinhard, Peter Sol Reinach, Szymon Ludwiczak, Huan Luo, Marah Hussain Omar Ba Salem, Miriam Monika Kraemer, Heike Biebermann, Vinodh Kakkassery, Stefan Mergler

**Affiliations:** 1 Klinik für Augenheilkunde, Charité – Universitätsmedizin Berlin, Corporate Member of Freie Universität Berlin, Humboldt-Universität zu Berlin and Berlin Institute of Health, Berlin, Germany; 2 Department of Cell Morphology and Molecular Neurobiology, Faculty of Biology and Biotechnology, Ruhr University Bochum, Bochum, Germany; 3 School of Ophthalmology and Optometry, Wenzhou Medical University, Wenzhou, PR China; 4 Institut für Experimentelle Pädiatrische Endokrinologie, Charité – Universitätsmedizin Berlin, Corporate Member of Freie Universität Berlin, Humboldt-Universität zu Berlin and Berlin Institute of Health, Berlin, Germany; 5 Universität zu Lübeck, Klinik für Augenheilkunde – Universitätsklinikum Schleswig-Holstein (Campus Lübeck), Lübeck, Germany

**Keywords:** Calcium signalling, Eye cancer, Patch clamp, Fluorescence imaging, Transient receptor potential channels

Correction to: *Laboratory Investigation*

10.1038/s41374-020-00485-2

Three different clarifications are warranted to better understand our findings and conclusions.

(1) On page 9 in the PDF version, subtitle “Medium acidification has less influence on Ca^2+^ regulation” should instead read:

Role of Asc-induced acidification in increasing Ca^2+^ influx

Section summary clarification: The role was reevaluated of Asc-induced medium acidification in contributing to increases in Ca^2+^ influx. We wish to clarify that the responses to Asc were much larger than those induced by medium acidification. Therefore, lowering the pH does not account for how Asc increases intracellular Ca^2+^ influx.

(2) Subtitle for Fig. 10 should be corrected as:

Acidifying-induced Ca^2+^ transients in etoposide-resistant and -sensitive WERI-Rb1 cells

(3) The following alteration is more precise regarding the role of TRPV1 channel activation during medium acidification. (page 11 - paragraph 2 and page 14 - paragraph 3; PDF version):

Section summary clarification: TRPV1 activation does not contribute to the small increases in Ca^2+^ influx resulting from medium acidification. This channel is not involved since the decline in pH occurred over a range known not to alter its activity. We suggest that mechanisms other than acidification are the main contributors underlying Asc–induced Ca^2+^ transients. Moreover, WERI-Rb1 cells possibly possess other pH sensitive pathways since lowering the pH induced significant increases in Ca^2+^ influx.

These corrections do not affect the results and conclusions of the article.

All authors agree with the corrections.

